# Achille beltrame: an exceptional wedding procession

**DOI:** 10.1007/s40618-025-02801-3

**Published:** 2026-01-08

**Authors:** Wouter W. de Herder

**Affiliations:** Erasmus MC, Department of Internal Medicine, Sector of Endocrinology, Rg 520, Dr. Molewaterplein 40, 3015 GD Rotterdam, The Netherlands

**Fig. 1:**
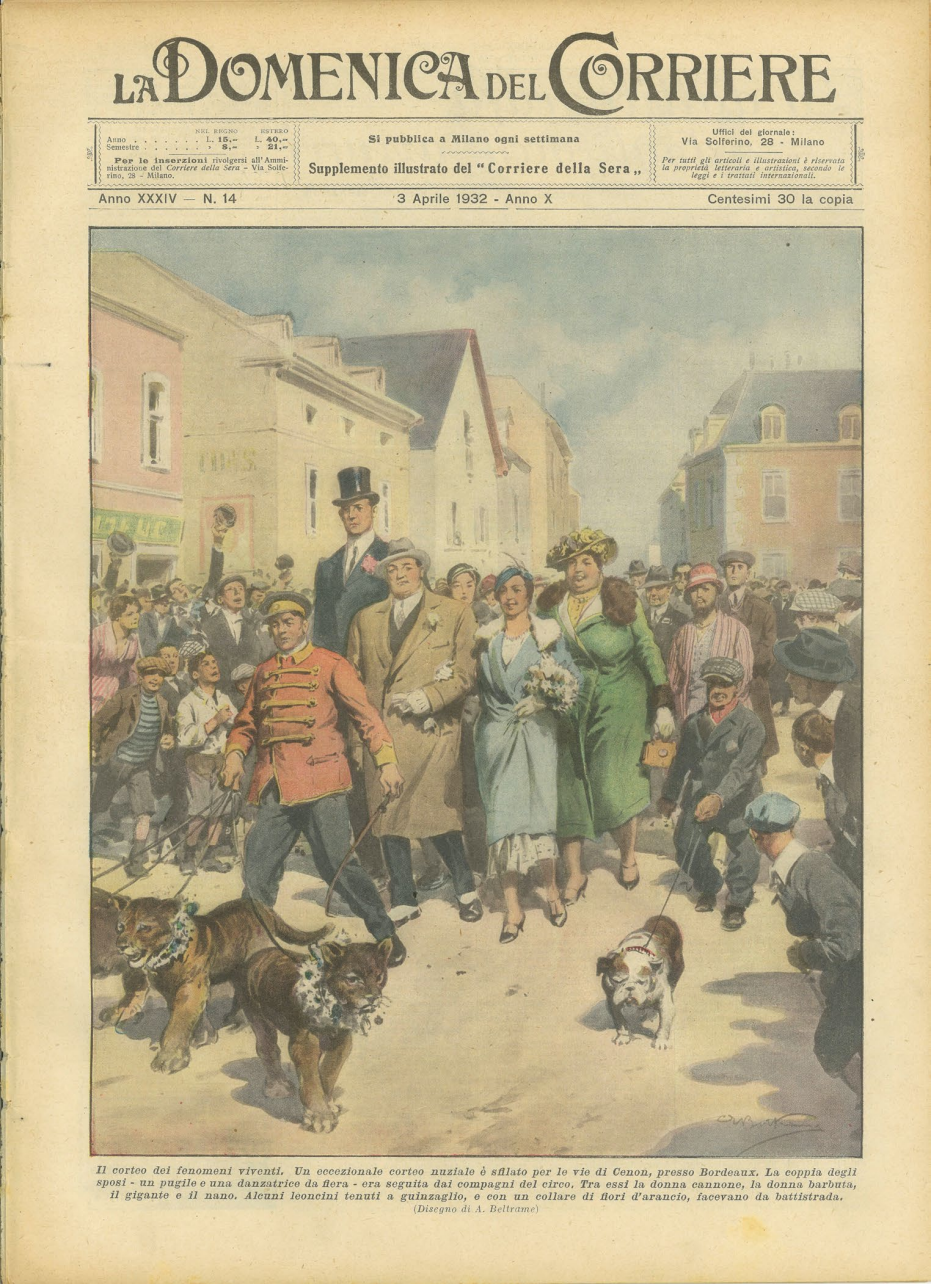
Front cover of “La Domenica del Corriere”, 3 April 1932

Front cover painting for the Italian weekly “La Domenica del Corriere” (The Sunday Courier) dating 3 April 1932 by Achille Beltrame, depicting an exceptional wedding procession in Cenon, Gironde, France (Fig. 1). The translated subtitle reads “*The procession of living phenomena. An exceptional wedding procession paraded through the streets of Cenon*,* near Bordeaux. The newlywed couple - a boxer and a dancer - were followed by their circus partners. Among them were the obese woman*,* the bearded woman*,* the giant and the midget. A few lion cubs*,* held on leashes or wearing collars of orange blossoms*,* led the way. (Drawing: A. Beltrame)*”.

Giants, small stature people, bearded women, strongmen, and morbidly obese men and women were popular characters in the circuses and sideshows, or fairs in pre-World War 2 Europe, the USA & Canada and Australia, where they appeared before an audience who usually paid to meet and greet them.

Unfortunately, it is unknown who the people in the procession in the Beltrame picture were: the tall man wearing a black top hat in the picture resembles the approximately 2.40 m. tall Italian acromegalic giant Almiro Crema (1900–1944 – also known as: “Gigante Golia”). Almiro Crema was married to the > 200 kg. weighing obese woman Térésina Alfonso/Alforno, who might be the lady in the green coat with fur collar in the picture [1]. Many small stature people were employed by the circuses and fairs in those days. The hirsute bearded woman in the pink outfit and wearing an orange-red hat at the right in the picture might have been the 1.65 m. tall bearded woman Clémentine Delait (1865–1939), who toured England & France in 1932. She ceased her commercial activities in 1934 [2].

Achille Beltrame (Arzignano, 18 March 1871–Milan, 19 February 1945) was a well-known Italian painter and illustrator. His name is closely tied to the covers which he produced for “La Domenica del Corriere”, appearing from the beginning of 1900 until 26 November 1944.

The picture/newspaper is from the collection of W.W. de Herder.
